# DEMENTIA CAREGIVERS’ ETHICAL CONSIDERATIONS OF HOME-MONITORING ASSISTIVE ROBOTS IN DEMENTIA CARE

**DOI:** 10.1093/geroni/igad104.3790

**Published:** 2023-12-21

**Authors:** Jing Wang, Dain LaRoche, Momotaz Begum, Xueting Tang, Sajay Arthanat

**Affiliations:** University of New Hampshire, Durham, New Hampshire, United States; University of New Hampshire, Durham, New Hampshire, United States; The University of New Hampshire, Durham, New Hampshire, United States; Fudan University, Shanghai, Shanghai, China (People’s Republic); The University of New Hampshire, Durham, New Hampshire, United States

## Abstract

Understanding dementia caregivers’ perspectives on the ethical dimensions of socially assistive robot technology is essential for its responsible and sustainable implementation. This study provides comprehensive insights into the ethical considerations of using home-monitoring assistive robots in dementia care, as seen through the perspectives of caregivers. A focus group and a follow-up interview were conducted with seven participant caregivers following their exploration of a home-monitoring assistive robot sharing their insights and experiences related to its use in dementia care. The data were subjected to thematic analysis, identifying recurring patterns and themes within the participants’ accounts. Key findings included Responsibility and Safety: Need for safety outweighed privacy concerns of caregivers. They highlighted features such as reminders, danger alerts, and integration with security systems; Autonomy and Informed Consent: our findings emphasized promoting robot technology while respecting the autonomy of individuals with dementia in deciding technology use for their own care, acknowledging the variability in their responses; Technology Maturity and Expectation Management: Caregivers expressed expectations regarding robot functionalities, highlighting the importance of technological reliability, setup processes, and technical support. Dilemma in Monitoring and Privacy: Striking a balance between monitoring and privacy emerged as a crucial concern, sparking discussions on camera usage, sensor placement, and maintaining a suitable equilibrium; Affordability Challenges: Caregivers recognized the affordability challenges associated with robot technology as a significant hurdle. The themes highlight the complexity of integrating Socially assistive robots for dementia care responsibly, thereby informing future development and implementation strategies to ensure its beneficial and ethical use.

